# The influence of abiotic factors on the bloom-forming alga *Ulva flexuosa* (Ulvaceae, Chlorophyta): possibilities for the control of the green tides in freshwater ecosystems

**DOI:** 10.1007/s10811-017-1301-5

**Published:** 2017-11-07

**Authors:** Andrzej Stanisław Rybak, Maciej Gąbka

**Affiliations:** Department of Hydrobiology, Institute of Environmental Biology, Faculty of Biology, Adam Mickiewicz University, Poznań, Umultowska st. 89, PL 61-614 Poznań, Poland

**Keywords:** Chlorophyta, Freshwater *Ulva*, *Enteromorpha*-like *Ulva*, Macroalgae bloom, Green tide, Nutrients

## Abstract

*Ulva* species are characterised by the capacity to achieve rapid biomass increase, which results in the formation of “green tides”, particularly in nutrient-rich seawaters. Over the last decade, formation of large-scale *Ulva* mats has been increasingly observed in freshwater systems in Central Europe. Mass development of *Ulva* in freshwater ecosystems presents a growing burden in spite of its economic benefits. This study explores the formation dynamics of *Ulva flexuosa* mats with respect to habitat conditions, using the examples of a number of water systems located in Poland. Elevated water temperature, pH and high concentration of sulphates are among the most important factors affecting biometric parameters of *Ulva* blooms. An evident disparity was observed between lotic water ecosystems and lentic water ecosystems, which differed in terms of chemical characteristics of the habitat and mat structure properties. In flowing water, *U. flexuosa* displays a definitely higher potential for blooms. On the other hand, mass occurrence of *U. flexuosa* in freshwaters is caused by the inflow of fecund waters, especially following intense precipitation in summertime, as well as by periodic increases in salinity, pH and sulphate levels. The study suggests that potential *U. flexuosa* blooms in landlocked ecosystems may be controlled by means of reducing the inflow of particularly sulphate-rich waters.

**Electronic supplementary material:**

The online version of this article (10.1007/s10811-017-1301-5) contains supplementary material, which is available to authorized users.

## Introduction

Macroalgae, in particular green algae of the genus *Ulva* (Ulvaceae, Chlorophyta), are distinguished by their capacity for rapid biomass increase over a short period of time (Fletcher 1996; Taylor et al. 2001). Large-scale occurrences of free-floating *Ulva* mats were observed as early as the 1970s in the eutrophic offshore waters of North and South America, Europe, Asia and Australia (Wang et al. 2016). Mass occurrence of *Ulva* species in marine waters is referred to as “green tide”, denoting the colouration of water due to large volume of algal thalli suspended in it (Gao et al. 2016). The green tides of *Ulva prolifera*, occurring annually in the Quingdao Gulf (Yellow Sea, China) since 2008, seem to be the most impressive phenomenon of this kind (Hu and He 2008; Hu et al. 2010; Duan et al. 2012). Persisting for a long period of time, *Ulva* mats have an impact on physical and chemical conditions in marine habitats (Van Alstyne et al. 2015) as well as on sea life found in such an environment (Smetacek and Zingone 2013). The impact of green tides manifests itself primarily by (i) local limitation of nitrogen availability for photoautotrophic organisms, (ii) control of daily fluctuations in pH, (iii) reduced oxygenation in demersal waters and (iv) production of allelopathic compounds (Van Alstyne et al. 2015). Large-scale green tides of *Ulva* in marine waters affect functional water quality and have a number of economic ramifications (Gravier et al. 2011; Smetacek and Zingone 2013).

*Ulva* species belong to a macroalgal group characterised by extensive tolerance in terms of key habitat parameters (such as e.g. temperature, light intensity, oxygenation, salinity and nutrients) (Fletcher 1996). The ability to adapt rapidly to variable environmental conditions, including high resistance to metal contamination, makes *Ulva* “physiologically hardy” (Hurd et al. 2014). The considerable resilience to extreme fluctuations in physical and chemical parameters, as observed in tidal waters, promotes consolidation of quick and effective mechanisms to achieve optimal reproductive quality (Fletcher 1996; Taylor et al. 2001).

Extensive green tides of opportunistic macroalgae are permanently monitored in the marine foreshore (Leliaert et al. 2009; Gravier et al. 2011). Current monitoring methods rely on the analysis of satellite imagery performed in time of free-floating and intertidal blooms of macroalgae (Nelson et al. 2003b), and modelling of green tide formation dynamics by means of field surveillance (Martins and Marques 2002; Perrot et al. 2014a; Xu et al. 2016). Meanwhile, methods which inhibit formation of green tides consist chiefly in wide-scale preventive operations, aimed to stem the run-off of nutrient-rich waters from agricultural areas. Reduction of nitrate and phosphate levels in rivers which discharge into seas is a particularly important measure (Kamer and Kennison 1995; De Casabianca et al. 2002; Perrot et al. 2014b).

Moreover, *Ulva* has been reported to form mats in freshwater systems (Messyasz and Rybak 2011; Rybak et al. 2014). A number of instances involving *U. flexuosa* have been observed in lakes, ponds and rivers in Central Europe (Mareš et al. 2011; Messyasz and Rybak 2011). The above studies, however, were concerned only with episodic and short-term occurrences of *U. flexuosa* in freshwater ecosystems (Rybak 2015). No research to date has addressed the dynamics of formation of large *U. flexuosa* blooms which would persist in freshwaters for a longer period of time; this shortage was the principal rationale for this study to be undertaken.

Cases of abundant presence of *U. flexuosa* in inland waters were investigated to obtain more information about the varied ecological aspects of green tides. Therefore, the main objective of our research was to analyse changes in physical and chemical parameters in freshwater ecosystems, including both lotic and lentic waters in which evident *U. flexuosa* blooms were observed. The composition and distribution of *U. flexuosa* blooms was analysed in detail, in order to examine the potential correlation between spatial changes in biogenic components and the occurrence of *U. flexuosa* mats. Also, our study aimed to determine the procedures which would decrease formation of large-scale *U. flexuosa* mats in freshwater ecosystems.

## Materials and methods

### Study species

The collected thalli of freshwater *Ulva* were identified to the species level using the ITS2 (internally transcribed spacer) region of rDNA (ribosomal deoxyribonucleic acid) and *rbc*L (Rubisco) gene sequences, as previously described by Rybak et al. (2014). Determination of species also relied on the examination of morphological characteristics as presented by Koeman and Van Den Hoek (1981), which was further supplemented by the measurement of cell wall thickness, circumferences of the cell and pyrenoid, as well diameter of the latter, according to suggestions in Rybak (2015). Identification keys devised by Huisman (2008) and taxonomical works by Hardy and Guiry (2003) were employed as well.

All specimens were determined to belong to the species *U. flexuosa* Wulfen. Following identification, its current taxonomic status was verified using AlgaeBase (Guiry and Guiry 2017).

### Research locations

**Fig. 1:**
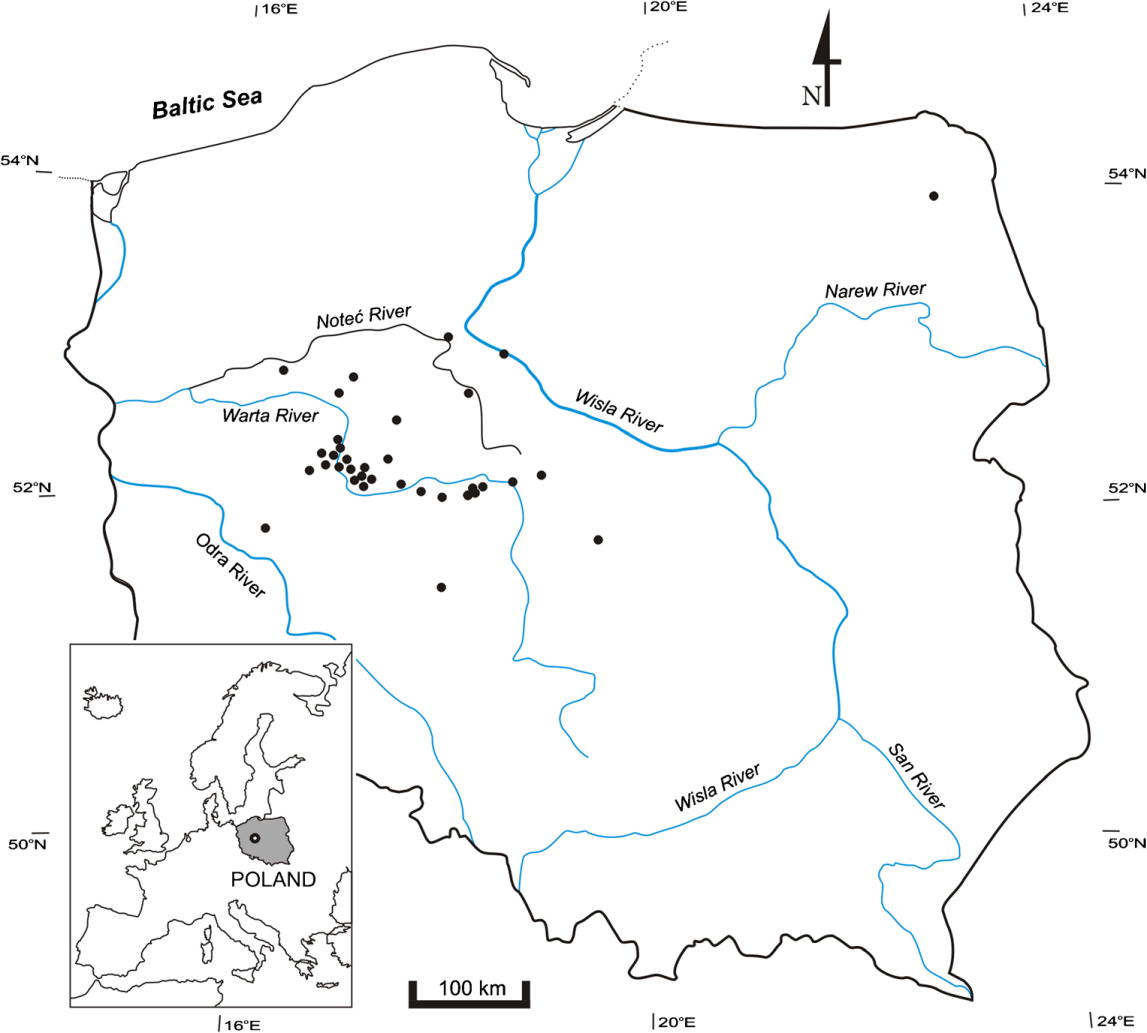
Distribution of sampling locations of freshwater *Ulva flexuosa* in Poland

The environmental study spanned freshwater *U. flexuosa* populations from 35 aquatic ecosystems located in Poland (Central Europe) (Fig. 1). Their origins varied, including natural, half-natural to entirely anthropogenic systems (Table S1). As regards the form, *U. flexuosa* populations were found in such water systems: ponds (9 populations), rivers (8), lakes (8), streams (3), rainwater tanks (3), oxbow lakes (2), a peat-bog (1), and a water canal (1).

### Field samples

**Fig. 2:**
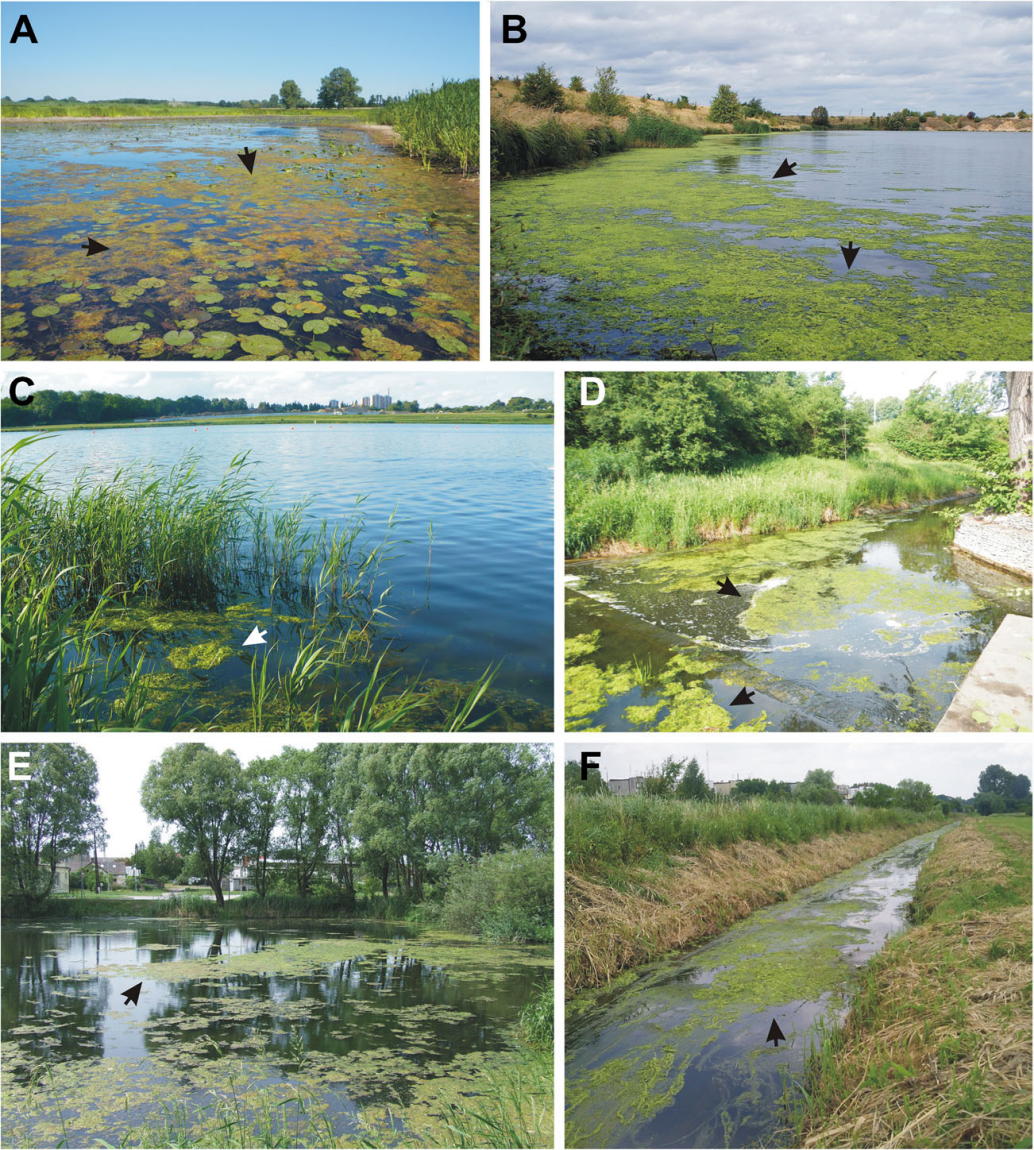
Examples of the “green tide” of *Ulva flexuosa* in freshwater ecosystems. Moderate bloom of *Ulva* in the Białobrzeg oxbow lake (**a**). Large-scale green tides along the shoreline of the Konin Pond (**b**). Green tides in the Malta Lake (**c**). Unattached forms of green-tide *Ulva* in the Cybina River (**d**). Free-floating *Ulva* thalli in the Tulce Pond (**e**). Massive floating green mats in the Nielba River (**f**). Photo credits: **a**, **d** Andrzej S. Rybak; **b** Sławomir Mielczarek; **c** Andrzej Woyda-Płoszczyca; **e** Violetta Socha; **f** Maciej Koperski

Sampling was performed when macroalgal blooms were present on the water surface (Fig. 2). At each site, samples of water and *U. flexuosa* thalli were collected simultaneously. In those cases where *Ulva* blooms persisted for a substantial period, the thalli were sampled repeatedly throughout the vegetation season (from April to September), in order to analyse the composition of the mat at several stages of its development (stage 1: mat formation; stage 2: optimal mat development; and stage 3: mat decline). Long-lasting green tides were analysed in 6 water ecosystems: the Malta Lake (34 samples), the Dworski Stream (27), the Michałówka Stream (25), the Nielba River (23), the Świątnica Stream (22), and the Tulce Pond (17). Mat structures as well as physical and chemical parameters in those ecosystems where blooms persisted for 1 to 2 weeks were analysed once (28 samples) or twice (2 samples). In total, researchers collected 178 samples, 104 of which originated from ecosystems with flowing water, whereas the remaining 74 samples were taken from lentic ecosystems (lakes and ponds).

Water was sampled by hand, directly from underneath the mats, using 1.0-L sterile plastic bottles (Roth, Germany). Long-sleeved veterinary-grade gloves were used in the process to prevent contamination. In lakes and ponds, water samples were taken from a rowboat, by means of which the mats were cautiously approached on the side exposed to the pelagic zone so as not to disturb sediments. Each water sample was filtered through a coarse plastic sieve to separate vascular plants and other filamentous macroalgae. Subsequently, water samples were placed in 0.5-L sterile plastic containers (Roth, Germany), preserved using 0.5 mL of chloroform (Sigma-Aldrich, Germany) and stored at 4 °C. At the laboratory, the samples were filtered through a nitrocellulose microbiological filter with a pore size of 0.45 μm and stored in a freezer at − 20 °C.

### Measurements

Plot locations were determined using a handheld Garmin Geographic Positioning System (model Oregon 550 t) and PVC markers. The percentage of water surface covered by *U. flexuosa* thalli (in a 2 × 2 m square), the number of thalli (in a 2 × 2 m square), as well as the density of thalli per cubic meter of the mats were evaluated in the field. The surface of macroalgal mats (m^2^) was plotted on a geo-referenced map using GIS (Arcview 3.0).

**Fig. 3:**
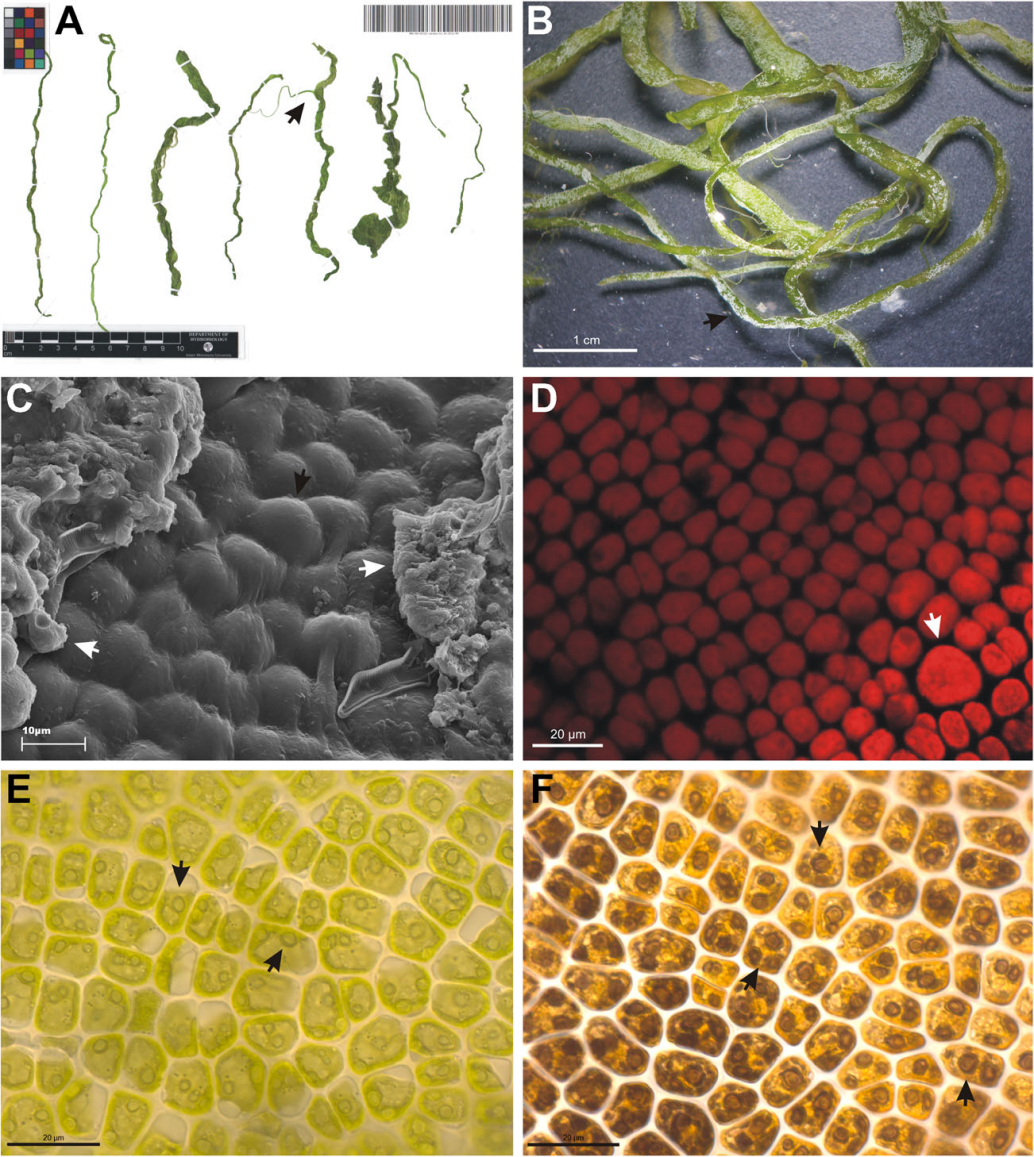
Thalli and cells of freshwater *Ulva flexuosa*. Herbarium sheet of *Ulva* with branched thallus (arrow) (**a**). Juvenile thalli with calcium carbonate crystals on the surface (arrow), as seen in stereoscopic microscopy (**b**). Surface of thalli with rounded cells (black arrow) and CaCO_3_ crystals (white arrows), as seen in scanning microscopy (**c**). Formation of proliferations from a swollen cell (arrow) in the central parts of the thallus, as seen in confocal microscopy (**d**). Cup-shaped chromatophore with 2–4 pyrenoids (arrows), as seen in light microscopy (**e**). Lugol’s iodine-stained pyrenoids (arrows) in the proximity of a chromatophore, as seen in light microscopy (**f**). Photo credits: **a**–**f** Andrzej S. Rybak

Laboratory analyses, including examination of thallus and cell morphology, were carried out using Zeiss Axioskop 2 MOT light microscope, Zeiss SteREO Discovery V20 stereoscope microscope, ZEISS LSM 700 confocal microscope and TESCAN Vega scanning microscope. Photographic documentation (from SEM (scanning electron microscope) and CLSM (confocal laser scanning microscopy)) was obtained at the Laboratory of Scanning Microscopy, Faculty of Biology, Adam Mickiewicz University in Poznań, Poland. Voucher specimens were sourced from the Natural History Collections at the Faculty of Biology (Poznań Algae Herbarium (POZA)) at the said university (Fig. 3).

Further analyses were concerned with physicochemical parameters of water samples obtained in all plots from the macroalgae contact zone; the parameters in question were collated with factors such as mat composition and depth. Water temperature, pH, total dissolved solids (TDS), conductivity (EC) and oxygen levels (DO) were measured using Professional Plus multi-parameter instrument (YSI, USA). Water samples were subsequently stored in a refrigerator at 4 °C; chemical analyses were performed by means of HACH DR 2800 spectrophotometer using standard hydrochemical methods. Concentrations of the following variables were determined: ammonium nitrogen (N-NH_4_), nitrate nitrogen (N-NO_3_), phosphate (P-PO_4_), sulphate (SO_4_), chloride (Cl), sodium chloride (NaCl) and total iron (Fe_total_).

### Data analysis

Ordination methods were used to establish relationships between physicochemical factors and *Ulva* blooms. Species–environment relationships of *U. flexuosa* were explored through redundancy analysis (RDA) (Legendre et al. 2012). Due to skewed distributions of most variables, square root transformation was performed. To reduce the number of variables, a forward selection procedure using Monte Carlo test with 999 permutations was applied (variables were discarded until significance threshold of *p* < 0.05 was reached). Variables with significance levels below *p* < 0.05 were passively projected into diagrams. Ordination was performed using the CANOCO software package (Gilliam and Saunders 2003).

A nonparametric Mann-Whitney test was applied to determine the significance of differences between the biometric characteristics and physicochemical parameters for all the sites. These statistical analyses were carried out using the R 3.0.1 statistical package (R Development Core Team 2013, using the vegan package; Viechtbauer 2010, Oksanen et al. 2011).

## Results

### Water chemistry

Waters containing *U. flexuosa* mats were typified by high levels of nutrients, conductivity and pH. This was accompanied by medium or low oxygen concentration (6.3 ± 2.8 mg O_2_ L^−1^). As regards nutrients, *Ulva* was found in habitats which were particularly rich in N-NH_4_ (0.58 ± 0.50 mg N L^−1^), P-PO_4_ (0.24 ± 0.31 mg P L^−1^) and displayed high value of conductivity (1009.9 ± 755.3 μS cm). Low concentrations of total iron were determined, especially in well-oxygenated habitats (0.17 mg ± 0.35 Fe L^−1^). Also, high concentrations of chloride (104.1 ± 75.9 mg Cl L^−1^) and sulphate (100.3 ± 42.9 mg SO_4_ L^−1^) were a salient feature of habitats where the blooms were encountered (Table 1).

**Table 1:** Average values of physicochemical properties of water for the examined sites and values of *Ulva* mat features (*n* = 178)

Parameters	Mean	Min.	Max.	SD
Percentage of thalli in 2 × 2 m square	59.92	5.00	100.00	29.46
Surface of the macroalgae mats (m^2^)	3.09	0.10	30.00	4.27
Number of thalli in 2 × 2 m square	64.68	4.00	215.00	35.11
Density of thalli in m^−2^ of the mats	122.49	0.65	1021.25	187.52
Temperature (°C)	20.13	11.30	27.90	3.94
DO (mg L^−1^)	6.29	0.25	11.85	2.81
Conductivity (μS cm^−1^)	1009.93	441.40	9873.00	755.29
TDS (mg L^−1^)	536.75	84.90	965.00	146.56
pH (−)	8.16	5.03	10.47	0.88
N-NO_3_ (mg L^−1^)	0.25	0.00	1.40	0.28
N-NH_4_ (mg L^−1^)	0.58	0.03	3.11	0.50
Fe_total_ (mg L^−1^)	0.17	0.00	2.40	0.35
P-PO_4_ (mg L^−1^)	0.24	0.00	1.65	0.31
Cl (mg L^−1^)	104.14	20.00	732.50	75.90
NaCl (mg L^−1^)	173.58	33.00	1208.60	127.35
SO_4_ (mg L^−1^)	100.28	25.00	198.00	42.88

**Fig. 4:**
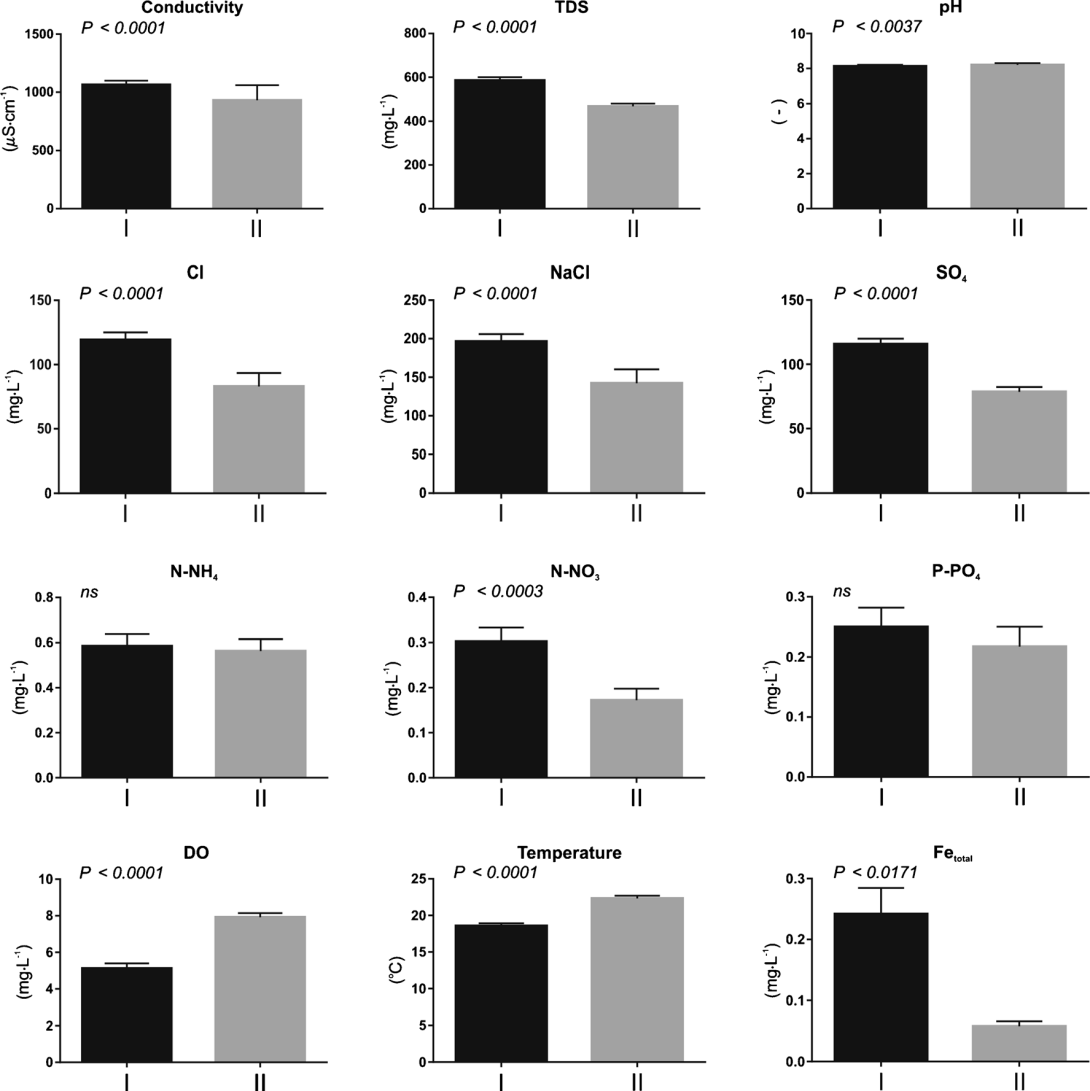
Results of Mann-Whitney test of differences in mean values of physicochemical properties between ecosystems with flowing (I) and stagnant waters (II), where ns denotes statistically not significant results

All test beds where *U. flexuosa* formed mats may be classified into two groups, namely those located in lentic waters and in lotic waters. Such division of habitats is dictated by chemical parameters, which in their turn depend on the degree of water mineralization and salinity. Lotic ecosystems demonstrated statistically significant (*p* < 0.001) higher levels of water mineralization (expressed in values for electrolytic conductivity and TDS volume), Cl concentration, SO_4_, N-NO_3_ and Fe_total_ in comparison with the lentic ecosystems. On the other hand, water temperature, DO and pH levels were higher (*p* < 0.005/0.001) in lentic waters than in the lotic habitats. No statistically significant differences between the groups were found with respect to P-PO_4_ and N-NH_4_ concentrations (Fig. 4).

### Variation in mat development

The formation of *U. flexuosa* mats in freshwater ecosystems is exceptionally dynamic. In numerous instances, mats would appear for a very brief period, decomposing entirely and disappearing from the surface of the waterbody or watercourse within several days. Sustained *U. flexuosa* blooms, i.e. those persisting in the established test beds for a number of weeks, demonstrated fluctuating changes in mat surface formation (over time). Under moderate and variable weather conditions, *U. flexuosa* reaches optimal development at the end of June and first days of July, continuing until early September. However, that period, during which blooms extend over the largest area of water surface, is different for individual ecosystems. Most often, *U. flexuosa* blooms developed in early summer (late June–early July) and towards the end of the season (August/September).

**Fig. 5:**
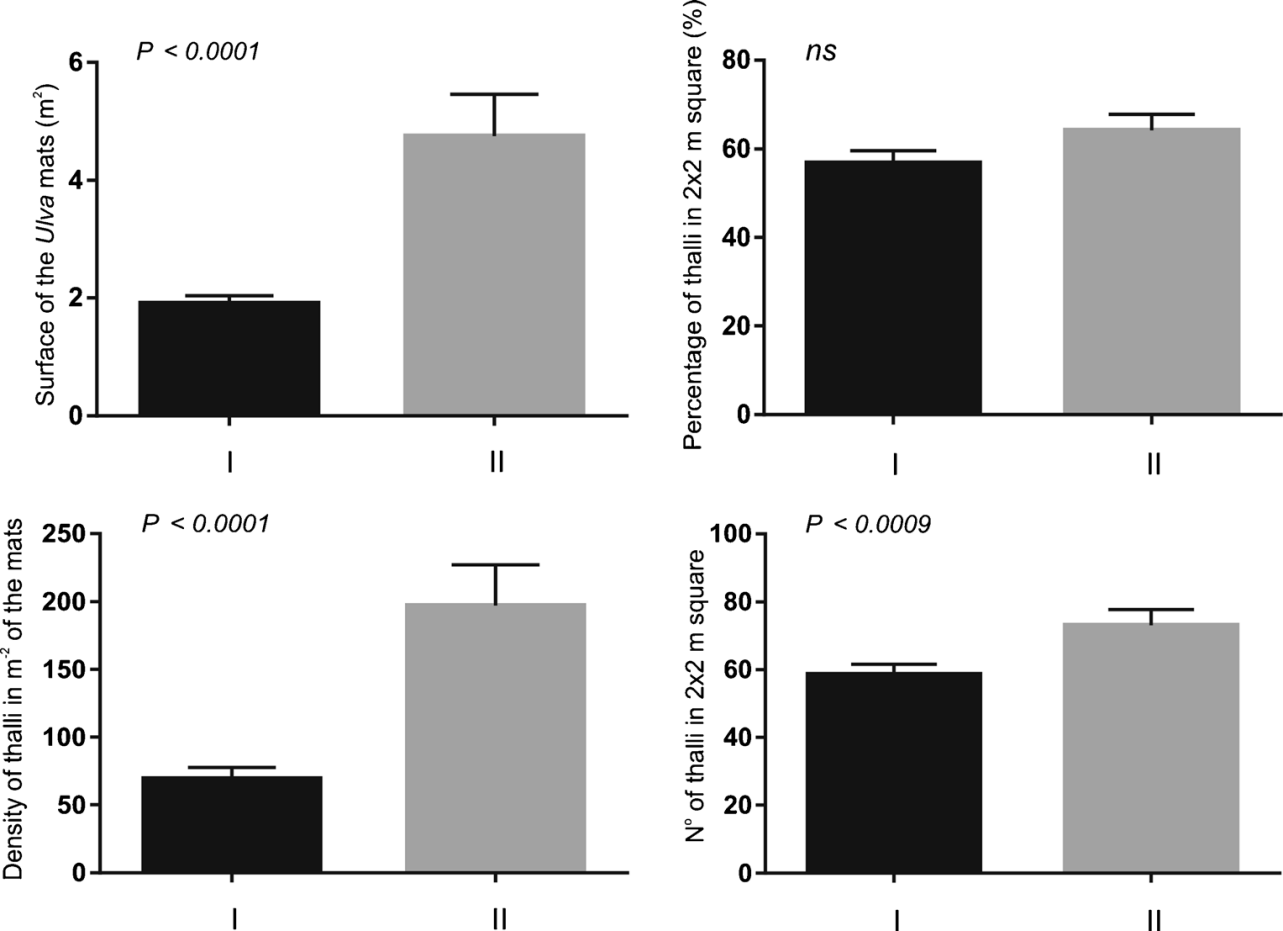
Results of Mann-Whitney test of differences in mean values of *Ulva* mat features between ecosystems with flowing (I) and stagnant waters (II), where ns denotes statistically not significant results

Significant differences were found between the properties of mats formed by *U. flexuosa* in the two groups of ecosystems distinguished on the basis of habitat parameters. Structural differences were statistically significant in the case of three analysed parameters (*p* < 0.0001). In the lotic ecosystems, mats covered a larger surface while their thalli were more numerous and more compact compared with lentic habitats (Fig. 5, Table 1). No statistically significant (*p* > 0.05) differences in the percentages of mat-forming thalli were found between the two habitat groups.

### Blooms of freshwater *Ulva*—environmental correlations

**Fig. 6:**
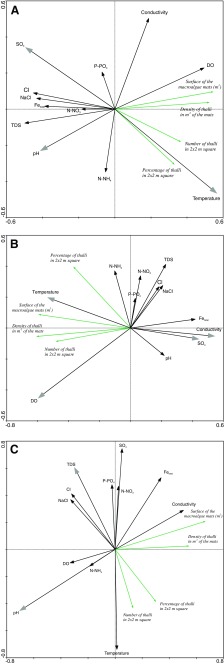
Biplots of redundancy analysis with features of *Ulva* mats for all sites (**a**). Variables which remained significant after selection (temperature, pH and sulphates) are marked with grey arrowheads. Plots with features of *Ulva* mats in sites with flowing waters (**b**). Variables which remained significant after selection (oxygen, temperature, conductivity and sulphates) are marked with grey arrowheads. Plots with features of *Ulva* mats in sites with stagnant waters (**c**). Variables which remained significant after selection (pH and TDS) are marked with grey arrowheads

The modelling based on the Monte Carlo test made it possible to identify the most important environmental variables which described the habitats of the studied *U. flexuosa* population. For all test beds, initial selection disclosed three major variables out of the 12 examined (*p* ≤ 0.05). The crucial physical and chemical parameters included water temperature, pH and sulphate concentration (Table 2). On the other hand, in a separate study into lotic habitats of *U. flexuosa*, the Monte Carlo test disclosed four major variables, namely water temperature, sulphate concentration as well as fluctuations in electrolytic conductivity and oxygen concentration. In our study, the analysis of lentic habitats yielded two alternative major parameters, i.e. changes in pH and TDS (*p* ≤ 0.05) (Table 2). The values obtained for environmental variables in the Monte Carlo test were different for each of the defined groups of habitats. Monte Carlo analysis confirmed that, given the considerable diversity and representative properties of *U. flexuosa* habitats, the characteristics of the niche would have to include the type of water flow (i.e. presence or absence of water flow). Figure 6 shows the relationship between physical and chemical parameters of water and structural features of the mats formed by a specific taxon in the compared ecosystems. In all analysed *U. flexuosa* test beds, i.e. both lotic and lentic macroalgal habitats, water temperature proved to be the main parameter affecting mat formation (Fig. 6a). The strongest correlation between the parameters defining *U. flexuosa* blooms and the degree of water oxygenation was determined for lotic water habitats (Fig. 6b). However, increase in pH was the principal factor affecting the development of *U. flexuosa* mats in lentic habitats (Fig. 6c).

**Table 2:** Results of the forward selection of environmental variables (Monte Carlo permutation test in RDA; *p* < 0.05 and *p* < 0.001 are statistically significant and provided in italics) in all samples, with division into two separate groups of samples (ecosystems): flowing and stagnant waters

Parameters	All sites			Flowing waters			Stagnant waters		
	λ	*F*	*P*	λ	*F*	*P*	λ	*F*	*P*
Temperature (°C)	0.08	14.64	*0.001*	0.06	7.49	*0.004*	0.01	0.69	0.422
pH (−)	0.06	12.06	*0.001*	0.00	0.56	0.485	0.13	10.78	*0.002*
SO_4_ (mg L^−1^)	0.03	7.97	*0.004*	0.03	4.83	*0.026*	0.02	1.80	0.150
TDS (mg L^−1^)	0.03	6.29	0.009	0.01	2.06	0.132	0.07	5.73	*0.011*
Conductivity (μS cm^−1^)	0.03	6.08	0.043	0.06	6.70	*0.004*	0.02	1.88	0.149
Cl (mg L^−1^)	0.01	2.06	0.147	0.02	2.60	0.086	0.00	0.04	0.926
DO (mg L^−1^)	0.01	1.86	0.178	0.11	13.23	*0.001*	0.02	1.77	0.182
N-NH_4_ (mg L^−1^)	0.00	0.89	0.368	0.01	0.56	0.526	0.00	0.12	0.819
Fe_total_ (mg L^−1^)	0.00	0.59	0.454	0.01	0.93	0.350	0.01	1.36	0.238
P-PO_4_ (mg L^−1^)	0.01	0.32	0.631	0.02	2.20	0.135	0.01	0.51	0.506
N-NO_3_ (mg L^−1^)	0.00	0.23	0.729	0.00	0.45	0.555	0.00	0.41	0.537
NaCl (mg L^−1^)	0.00	0.03	0.943	0.01	1.91	0.155	0.00	0.03	0.949

## Discussion

### The “green tides” of *Ulva*

Long-term studies into green tides of marine populations of *Ulva* have revealed a complex set of causes behind the phenomenon. Most often, water eutrophication is identified as the main factor inducing the formation of large-area blooms. It is nevertheless noted that in this very case, the process should be understood as “an acceleration of chemical inputs that favour photosynthesis and influence algal populations” (Fletcher 1996). Our analysis of *U. flexuosa* test beds demonstrated higher concentrations of nitrogen, yet the primary triggers of bloom included elevated water temperature, pH and sulphate concentration. Similarly, Mareš et al. (2011) indicated a considerable impact of sulphates on the occurrence of *Ulva* in Czech freshwater ecosystems. High pH levels reported for stagnant waters and high oxygen levels for fast-flowing water could be a consequence of high photosynthetic rates of the species. Higher pH values in sites with greater abundance of *Ulva* should probably be attributed to the high uptake of carbon (CO_2_) from the water. In literature, a reaction of this kind was demonstrated on the example of marine alga, *Gracilaria tenuistipitata* (Israel et al. 1999, 1992). Also, this phenomenon has been described for intertidal pools inhabited by marine populations of tubular *Ulva*, where elevated pH was observed on sunny days (Beer and Israel 1990; Björk et al. 2004).

Our study contributes the observation that presence or absence of water flow plays an important role for inland populations of *U. flexuosa*, as the process of bloom formation is clearly different in these two types of systems. In river systems, formation of dense mats of *U. flexuosa* depends heavily on changes in water temperature and oxygenation. Water movement and fluctuations in water level are physical processes which have an impact on the growth of thalli and the development of macroalgal blooms (Morand and Briand 1996; Msuya and Neori 2008). Water motion is a key factor of marine proliferation of macroalgae, influencing physiological rates and species composition directly or indirectly (Hurd 2000; Piñón-Gimate et al. 2008). The waterflow in the rivers and streams examined in this study may have stimulated the development of *U. flexuosa*, in that it resembled the wave and tidal mechanics of marine ecosystems (Hurd 2000; Hurd et al. 2014).

Algae of the genus *Ulva* are widely used in environmental monitoring (Morand and Briand 1996). Cosmopolitan species are employed in the monitoring of waters contaminated with metals (Rybak et al. 2012a, 2012b, 2013) and used to enrich biomass with microelements (Schroeder et al. 2013; Michalak and Chojnacka 2015). Mass development of *Ulva* species in waters containing high concentrations of nitrogen and phosphorus resulted in their being recommended as bioindicators of water contamination with nutrients (Morand and Briand 1996; Riddin and Adams 2010). As regards freshwater populations of *U. flexuosa*, utilisation of this species as an indicator of increased sulphate concentration seems reasonable. It should be noted, however, that some inland taxa, such as *U. flexuosa* subsp*. paradoxa*, occur only in extremely saline waters (e.g. watercourses and ponds adjacent to motorways). Their domination in such habitats signifies anthropogenic contamination following chemical enrichment treatments using mineral salts (Rybak et al. 2014).

The green tides of micro- and macroalgae are a worldwide problem, notably in maritime countries with a well-developed fishery, aquaculture and tourism (Han et al. 2013; Śliwińska-Wilczewska et al. 2016). High-resolution satellite photography provides valuable data on the scale and intensity of this phenomenon (Zhang et al. 2013, 2015; Gao et al. 2016). Analysis of satellite imagery carried out by means of the Moderate Resolution Imaging Spectroradiometer (MODIS) enables precise identification of the sites where *Ulva* forms blooms and permits directional tracing of their displacement, either along the coast or from the open sea towards its littoral zone. Unfortunately, this method is unreliable in the case of blooms of benthic macroalgae (Liu et al. 2009; Hu et al. 2010; Pang et al. 2010).

Forecasting marine *Ulva* blooms relies strongly on mathematical modelling and analysis of population development (Martins and Marques 2002; Alström-Rapaport et al. 2010; Hu et al. 2010; Perrot et al. 2014a). Most models describing growth of opportunistic *Ulva* species which form blooms in tidal waters and estuaries involve such factors as light, temperature, salinity and concentrations of nitrogen and phosphorus. Significantly enough, models generated in this manner cannot be universally applied but may be used only in the regions where they have been developed and tested (Martins and Marques 2002; Aldridge and Trimmer 2009; Perrot et al. 2014a; Xu et al. 2016). Moreover, even very detailed predictions of *Ulva* growth rates for estuaries fail to prove true in winter and early summer months. Their invalidity is due to the fact that the algorithm omits a number of unstable variables, such as (i) extreme conditions (decreased salinity in winter and increased temperature in summer), and (ii) individual physiological response of a macroalga, including e.g. sporulation or desiccation (Martins and Marques 2002; Giardino et al. 2015).

The optimal period of development of *Ulva* species in natural and anthropogenic habitats (fish ponds), i.e. the period when the largest biomass is achieved, is in spring and during warm summer months (Hernández et al. 2008). Monitoring demonstrates that biomass proliferation is positively correlated with the length of day and water temperature; also, it may be more intensive at a depth exceeding 1 m than on the surface (Nelson et al. 2003a). Freshwater *U. flexuosa* have been observed to develop as generations which (i) are entirely submerged and attached to a solid substrate, (ii) trail on a sandy bed and (iii) drift freely in the form of mats. However, the drifting mat is the predominant form, occurring most often and producing the most abundant biomass. In this study, only isolated thalli of *U. flexuosa* could be found at the bottom whereas one square metre of a drifting mat was composed of as many as several hundred thalli. In the inland systems, the thalli of *U. flexuosa* formed the most extensive mats during warm summer months, similarly to marine populations. The thalli which combine into a dense macroalgal mat differ from one another in terms of size and colour. In this study, the thalli in the central section of the mat and those forming its bottom layer were dark green while in the outer layers they were light green. Studies concerned with marine green tides confirm that algae condensed into a mat can adapt to poor light conditions by increasing their chlorophyll content (Malta et al. 2003). Consequently, the varied colouration of individual mat layers reflects the response to changing light gradient.

### Impacts of large-scale *Ulva* development

Large-scale *Ulva* blooms have an impact on the functioning of aquatic organisms (Charlier et al. 2008; Smetacek and Zingone 2013). Studies of sites with extensive blooms of *Ulva intestinalis*, *U. compressa* and *U. lactuca* revealed a decrease in the density of invertebrate populations (Bolam and Fernandes 2002). The green tides of *Ulva* substantially reduce the occurrence of the bottom fauna, thus diminishing the food base available to the marine macrofauna (Wan et al. 2017). Likewise, plants and animals in inland waters are seldom encountered in the areas dominated by populations of *U. flexuosa*. Nevertheless, in freshwater populations of *U. flexuosa*, increased population density of snails found in the mats was observed. The inner space of the tubular thalli of *U. flexuosa* provides both a refuge and a feeding site for *Lymnaea stagnalis* (Rybak 2016b; Rybak and Gąbka 2017). Potentially positive responses of aquatic organisms to mass occurrences of *Ulva* in freshwater ecosystems are also a matter to be thoroughly investigated in the future.

Considerable volumes of *Ulva* biomass are more than likely to affect the assets of the littoral areas in terms of tourist appeal (Charlier et al. 2008). The deposition of rotten thalli on the beaches should be immediately taken care of, considering the ensuing release of hydrogen sulphide, carbon disulphide and methyl sulphide (Wan et al. 2017). Mechanical or manual collection of *Ulva* biomass from the beaches as well as its management are very expensive (Gravier et al. 2011). Mass development of *Ulva* in freshwater ecosystems used as communication routes impedes the mobility of most waterborne craft (Rybak 2016a). Also, a considerable *Ulva* biomass was found to hamper the operations of small hydroelectric power stations located on small watercourses. According to Polish law, mass occurrence of macroalgae at seaside, lakeside or riverine resorts is the basis for excluding such public places from operational use (Rybak 2016a). Although *Ulva* blooms in freshwater ecosystems do not cover such extensive areas as is the case with marine waters, the thalli propelled by the wind towards the shore will form a long-shore bar. The accumulation of *Ulva* thalli deposited on the shores of a lake may considerably reduce the aesthetic assets of the beaches (especially in leisure resorts adjacent to any bodies of water), and therefore, it should be adequately handled. In view of the increasing number of reports concerning large-scale development of *Ulva* in lakes and rivers across Europe, effective methods of collecting and processing the problematic biomass should be devised.

### Conclusions

Large-scale blooms of *U. flexuosa* were encountered most often in substantially modified and intensively used water systems or watercourses which played the role of drainage basins collecting water combined with artificial fertilisers from agriculturally cultivated areas. The chief methods by means of which *Ulva* blooms in inland systems can be contained include the following: (1) strictly controlled, well-proportioned usage of chemical fertilisers on cultivated land; (2) protection of economically valuable watercourses and waterbodies against the inflow of nutrient-rich waters; (3) construction of barrier (buffer) systems in the shape of forested zones along the watercourses; and (4) construction of perimeter ditches and biological systems for water purification. Naturally, these methods constitute preventive rather than remedial measures.

We hope that our study will contribute to the store of knowledge concerning the ecological aspects of *Ulva* species in freshwaters and encourage further research into the biology and dynamics of development of these expansive macroalgae.

## Electronic supplementary material

Table S1 (DOCX 37 kb)
