# Supplementary material for: The influence of abiotic factors on the bloom-forming alga Ulva flexuosa (Ulvaceae, Chlorophyta): possibilities for the control of the green tides in freshwater ecosystems
Source: J Appl Phycol. 2017 Nov 7;30(2):1405–16. doi: 10.1007/s10811-017-1301-5 (PMC5928185; doi:10.1007/s10811-017-1301-5)
Supplement: Supplementary file 1 — (DOCX 37 kb) [file 10811_2017_1301_MOESM1_ESM.docx]

**Table S1** List of *Ulva flexuosa* sites included in analysis

|  | Ecosystem | Ecosystem name | Orgin | Locality | GPS | ATPOL | Herbarium  and  voucher N^o^ |
| --- | --- | --- | --- | --- | --- | --- | --- |
| 1 | pond | Junikowo Pond | anthropogenic | Poznań city,  Wielkopolska Region | N 52^o^ 22' 22,55''  E 16^o^ 51' 35,93'' | BD-08 | POZA-U0027 |
| 2 | river | Kiełbaska River | natural | Koło city,  Wielkopolska Region | N 52^o^ 12' 19,30''  E 18^o^ 30' 48,23'' | CD-29 | POZA-U0023 |
| 3 | river | Rgilewka River | natural | Grzegorzew city, Wielkopolska Region | N 52^o^ 12' 11''  E 18^o^ 44' 62'' | DD-21 | POZA-U0022 |
| 4 | pond | Rozlany Pond | anthropogenic | Poznań city,  Wielkopolska Region | N 52^o^ 22' 1,27''  E 16^o^ 51' 51,79'' | BD-08 | POZA-U0028 |
| 5 | river | Noteć River | natural | Szamocin city, Wielkopolska Region | N 53^o^ 04' 1,4''  E 17^o^ 03' 11,8'' | BC-29 | POZA-U0021 |
| 6 | pond | Arturówek Pond | anthropogenic | Łódź city,  Lodz Region | N 51° 49' 21,8 ''  E 19° 28' 27,3'' | DD-66 | POZA-U0152 |
| 7 | pond | Tulecki Pond | half-natural | Tulce village,  Wielkopolska Region | N 52^o^ 20' 35''  E 17^o^ 04' 40'' | BD-19 | POZA-U0019 |
| 8 | river | Nielba River | natural | Wągrowiec city, Wielkopolska Region | N 52^o^ 48' 7,41''  E 17^o^ 12' 31,43'' | CC-50 | POZA-U0024 POZA-U0025 |
| 9 | river | Samica Stęszewska River | natural | Stęszew city, Wielkopolska Region | N 52^o^ 17' 28,1''  E 16^o^ 41' 03,8'' | BD-17 | POZA-U0063 |
| 10 | lake | Laskownickie Lake | natural | Gołańcz city, Wielkopolska Region | N 52^o^ 54' 55,0''  E 17^o^ 15' 41,8'' | CC-41 | POZA-U0020 |
| 11 | oxyblow | non name | natural | Kórnik city,  Wielkopolska Region | N 52^o^ 12' 02,1''  E16^o^ 59' 40,5'' | BD-29 | POZA-U0026 |
| 12 | river | Kopla River | natural | Kórnik city,  Wielkopolska Region | N 52^o^ 20' 04,4''  E 17^o^ 03' 59,2'' | BD-19 | POZA-U0002 |
| 13 | river | Struga Średzka River | half-natural | Środa Wielkopolska city, Wielkopolska Region | N 52^o^ 13' 43,9''  E 17^o^ 14' 28,3'' | CD-20 | POZA-U0004 |
| 14 | rainwater tank | non name | anthropogenic | Września city, Wielkopolska Region | N 52^o^ 18' 47,8''  E 17^o^ 32' 28,4'' | CD-13 | POZA-U0009 |
| 15 | pond | non name | anthropogenic | Poznań city,  Wielkopolska Region | N 52^o^ 21' 40,1''  E 16^o^ 54' 46,6'' | BD-08 | POZA-U0010 |
| 16 | oxyblow | Port Drzewny | half-natural | Toruń city, Kuyavian-Pomeranian Region | N 52^o^ 00' 54,1''  E 18^o^ 29' 29'' | CE-69 | POZA-U0016 |
| 17 | lake | Pątnowskie Lake | natural | Konin city,  Wielkopolska Region | N 52° 18' 05.6 ''  E 18° 16' 34.9'' | CD-18 | POZA-U0083 POZA-U0084 |
| 18 | lake | Gosławskie Lake | natural | Konin city,  Wielkopolska Region | N 52° 17' 17.9 ''  E18° 12' 45.2'' | CD-17 | POZA-U0085 POZA-U0086 |
| 19 | lake | Licheńskie Lake | natural | Ślesin city,  Wielkopolska Region | N 52° 20' 21.2''  E 18° 21' 28.7'' | CD-18 | POZA-U0087 |
| 20 | stream | Świątnica Stream | natural | Poznań city,  Wielkopolska Region | N 52^o^ 21' 37''  E 17^o^ 02' 40'' | BD-09 | POZA-U0012 POZA-U0015 |
| 21 | stream | Michałówka Stream | natural | Kleszczewo city, Wielkopolska Region | N 52^o^ 20' 20''  E 17^o^ 02' 44'' | BD-19 | POZA-U0014 |
| 22 | lake | Malta Lake | anthropogenic | Poznań city,  Wielkopolska Region | N 52^o^ 24' 10,5''  E 16^o^ 57' 45,2'' | BD-09 | POZA-U0032 |
| 23 | stream | Dworski Stream | natural | Poznań city,  Wielkopolska Region | N 52^o^ 20' 20,4''  E 17^o^ 02' 10,1'' | BD-19 | POZA-U0008 POZA-U0082 |
| 24 | canal | Bydgowski Canal | anthropogenic | Bydgoszcz city, Kuyavian-Pomeranian Region | N 53° 08' 13,0''  E 17° 58' 11,8'' | CC-25 | POZA-U0096 |
| 25 | pond | Śródecki Pond | half-natural | Śródka village, Wielkopolska Region | N 52^o^ 17' 45,9''  E 17^o^ 07' 06,1'' | CD-10 | POZA-U0005 |
| 26 | rainwater tank | non name | anthropogenic | Kleszczewo village, Wielkopolska Region | N 52^o^ 18' 25,8''  E 17^o^ 10' 11,2'' | CD-10 | POZA-U0006 |
| 27 | peat-bog | non name | anthropogenic | Środa Wielkopolska city, Wielkopolska Region | N 52 ^o^ 12' 57,9''  E 17^o^ 16' 47,79'' | CD-21 | POZA-U0007 |
| 28 | lake | Lednica Lake | natural | Lubowo village, Wielkopolska Region | N 52^o^ 30' 40,18''  E 17^o^ 22' 30,46'' | CC-91 | POZA-U0011 POZA-U0012 POZA-U0080 |
| 29 | rainwater tank | non name | anthropogenic | Kleszczewo village, Wielkopolska Region | N 52^o^ 18' 34,4''  E 17^o^ 14' 07,3'' | CD-10 | POZA-U0090 |
| 30 | pond | Moraski Pond | half-natural | Poznań city,  Wielkopolska Region | N 52° 28' 03,8''  E 16° 55' 58,2'' | BC-98 | POZA-U0097 |
| 31 | river | Ołobok River | natural | Nowe Skalmierzyce city, Wielkopolska Region | N 51° 39' 32,9''  E 17° 56' 31,5'' | CD-85 | POZA-U0115 |
| 32 | pond | non name | anthropogenic | Konin city,  Wielkopolska Region | N 52^o^ 19' 19,4''  E 18^o^ 16' 16'' | CD-18 | POZA-U0116 |
| 33 | lake | Wigierskie Białe Lake | natural | Suwałki city,  Podlaskie region | N 54° 2' 12''  E 23° 5' 57'' | FB-19 | POZA-U0117 |
| 34 | lake | Brenno Lake | natural | Wijewo city, Wielkopolska Region | N 51° 55' 36,91''  E 16° 12' 38,62'' | BD-53 | POZA-U0118 |
| 35 | pond | non name | anthropogenic | Jacewo village, Kuyavian-Pomeranian Region | N 52° 48' 1,78''  E 18° 17' 14,23'' | CC-68 | POZA-U0122 |

ATPOL (**At**las of **Pol**and): grid square system for plant cartography.
